# Non-coding RNA: what is functional and what is junk?

**DOI:** 10.3389/fgene.2015.00002

**Published:** 2015-01-26

**Authors:** Alexander F. Palazzo, Eliza S. Lee

**Affiliations:** Department of Biochemistry, University of TorontoToronto, ON, Canada

**Keywords:** Junk RNA, Junk DNA, non-coding RNA, evolution, genome biology

## Abstract

The genomes of large multicellular eukaryotes are mostly comprised of non-protein coding DNA. Although there has been much agreement that a small fraction of these genomes has important biological functions, there has been much debate as to whether the rest contributes to development and/or homeostasis. Much of the speculation has centered on the genomic regions that are transcribed into RNA at some low level. Unfortunately these RNAs have been arbitrarily assigned various names, such as “intergenic RNA,” “long non-coding RNAs” etc., which have led to some confusion in the field. Many researchers believe that these transcripts represent a vast, unchartered world of functional non-coding RNAs (ncRNAs), simply because they exist. However, there are reasons to question this Panglossian view because it ignores our current understanding of how evolution shapes eukaryotic genomes and how the gene expression machinery works in eukaryotic cells. Although there are undoubtedly many more functional ncRNAs yet to be discovered and characterized, it is also likely that many of these transcripts are simply junk. Here, we discuss how to determine whether any given ncRNA has a function. Importantly, we advocate that in the absence of any such data, the appropriate null hypothesis is that the RNA in question is junk.

## INTRODUCTION

Starting with the discovery of transfer RNA and ribosomal RNA in the 1950s, non-coding RNAs (ncRNAs) with biological roles have been known for close to 60 years. Even in the late 1970s and early 1980s the existence of other functional ncRNAs was known, including RNAse P (Stark et al., 1978), snRNAs (Yang et al., 1981), and 7SL [the RNA component of the signal recognition particle (Walter and Blobel, 1982)]. Later, ncRNAs that serve to regulate chromosome structure, such as Xist, were discovered (Brockdorff et al., 1992). Since then, the number of new and putative functional ncRNAs has greatly expanded (for reviews see Wilusz et al., 2009; Wang and Chang, 2011; Ulitsky and Bartel, 2013; Rinn and Guttman, 2014). Interest in this field was further stimulated by the finding that almost all of the mammalian genome is transcribed at some level (Carninci et al., 2005; Birney et al., 2007; Djebali et al., 2012), with some individuals speculating that much of this pervasive transcription is likely functional (Mattick et al., 2010; Ecker et al., 2012; Pennisi, 2012). This idea was epitomized by the ENCODE consortium, which claimed to have assigned “biochemical functions for 80% of the genome” (ENCODE Project Consortium et al., 2012). Others have disagreed, pointing out that the vast majority of these novel transcripts are present at low levels, and that the term “function” had been misappropriated (Eddy, 2012; Doolittle, 2013; Graur et al., 2013; Niu and Jiang, 2013; Palazzo and Gregory, 2014). Despite these criticisms, the idea that the pervasive transcription of the human genome plays some role in homeostasis and/or development persists, with one group even proclaiming that they had “refuted the specific claims that most of the observed transcription across the human genome is random” (Mattick and Dinger, 2013).

At present, the distinction between functional ncRNAs and junk RNA appears to be quite vague. There has been, however, some effort to differentiate between these two groups, based on various criteria ranging from their expression levels and splicing to conservation. Ultimately these efforts have failed to bring consensus to the field.

A similar problem has plagued the investigation of whether transposable elements (TEs), which make up a significant proportion of most vertebrate genomes, have been exapted for the benefit of the host organism. Although some have claimed that many TEs are functional, a few groups have offered a much more balanced view that is in line with our current understanding of molecular evolution (de Souza et al., 2013; Elliott et al., 2014).

In this article we explain several concepts that researchers must keep in mind when evaluating whether a given ncRNA has a function at the organismal level. Importantly, the presence of low abundant non-functional transcripts is entirely consistent with our current understanding of how eukaryotic gene expression works and how the eukaryotic genome is shaped by evolution. With this in mind, researchers should take the approach that an uncharacterized non-coding RNA likely has no function, unless proven otherwise. This is the null hypothesis. If a given ncRNA has supplementary attributes that would not be expected to be found in junk RNA, then this would provide some evidence that this transcript may be functional.

## THE AMOUNT OF VARIOUS RNA SPECIES IN THE TYPICAL EUKARYOTIC CELL

As is evident from a number of sources, almost all of the human genome is transcribed. However, one must not confuse the number of different *types* of transcripts with their *abundance* in a typical cell. Many of the putative functional ncRNAs are present at very low levels and thus unlikely to be of any importance with respect to cell or organismal physiology. Importantly, the abundance of an ncRNA species roughly correlates with its level of conservation (Managadze et al., 2011), which is a good proxy for function (Doolittle et al., 2014; Elliott et al., 2014; however, see below); thus, determining the relative abundance of a given ncRNA in the relevant cell type is an important piece of information. However, one should keep in mind that if the ncRNA has catalytic activity or if it acts as a scaffold to regulate chromosomal architecture near its site of transcription, the RNA may not need to be present at very high levels to be able to perform its task.

**FIGURE 1:**
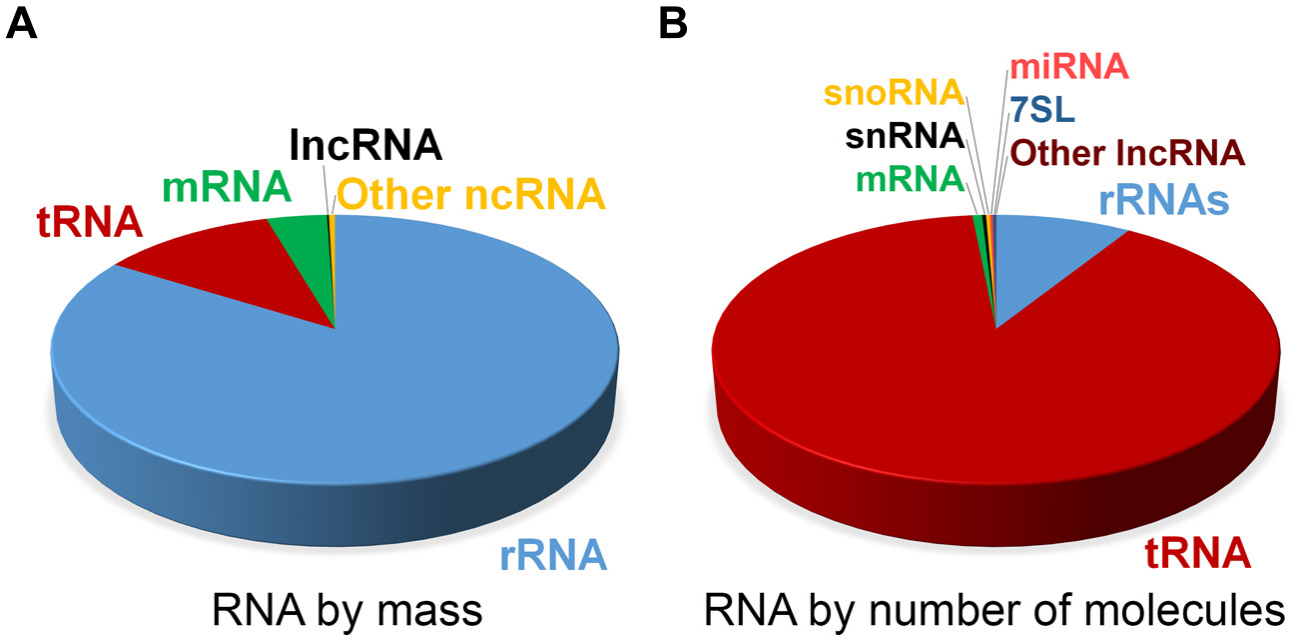
**Estimate of RNA levels in a typical mammalian cell.** Proportion of the various classes of RNA in mammalian somatic cells by total mass **(A)** and by absolute number of molecules **(B)**. Total number of RNA molecules is estimated at roughly 10^7^ per cell. Other ncRNAs in **(A)** include snRNA, snoRNA, and miRNA. Note that due to their relatively large sizes, rRNA, mRNA, and lncRNAs make up a larger proportion of the mass as compared to the overall number of molecules.

At steady state, the vast majority of human cellular RNA consists of rRNA (∼90% of total RNA for most cells, see **Table 1** and **Figure 1**). Although there is less tRNA by mass, their small size results in their molar level being higher than rRNA (**Figure 1**). Other abundant RNAs, such as mRNA, snRNA, and snoRNAs are present in aggregate at levels that are about 1–2 orders of magnitude lower than rRNA and tRNA (**Table 1** and **Figure 1**). Certain small RNAs, such as miRNA and piRNAs can be present at very high levels; however, this appears to be cell type dependent.

**Table 1:** Estimates of total RNA content in mammalian cells.

Type	Percent of total RNA by mass	Molecules per cell	Average size (kb)	Total weight picograms/cell	Notes	Reference
rRNAs	80 to 90	3–10 × 10^6^ (ribosomes)	6.9	10 to 30		Blobel and Potter (1967); Wolf and Schlessinger (1977), Duncan and Hershey (1983)
tRNA	10 to 15	3–10 × 10^7^	<0.1	1.5 to 5	About 10 tRNA molecules /ribosome	Waldron and Lacroute (1975)
mRNA	3 to 7	3–10 × 10^5^	1.7	0.25 to 0.9		Hastie and Bishop (1976); Carter et al. (2005)
hnRNA (pre-mRNA)	0.06 to 0.2	1–10 × 10^3^	10*	0.004 to 0.03	Estimated at 2–4% of mRNA by weight	Mortazavi et al. (2008); Menet et al. (2012)
Circular RNA	0.002 to 0.03	3–20 × 10^3^	∼0.5	0.0007 to 0.005	Estimated at 0.1–0.2% of mRNA**	Salzman et al. (2012); Guo et al. (2014)
snRNA	0.02 to 0.3	1–5 × 10^5^	0.1–0.2	0.008 to 0.04		Kiss and Filipowicz (1992); Castle et al. (2010)
snoRNA	0.04 to 0.2	2–3 × 10^5^	0.2	0.02 to 0.03		Kiss and Filipowicz (1992); Cooper (2000), Castle et al. (2010)
miRNA	0.003 to 0.02	1–3 × 10^5^	0.02	0.001 to 0.003	About 10^5^ molecules per 10 pg total RNA	Bissels et al. (2009)
7SL	0.01 to 0.2	3–20 × 10^4^	0.3	0.005 to 0.03	About 1–2 SRP molecules/100 ribosomes	Raue et al. (2007); Castle et al. (2010)
Xist	0.0003 to 0.02	0.1–2 × 10^3^	2.8	0.0001 to 0.003		Buzin et al. (1994); Castle et al. (2010)
Other lncRNA	0.03 to 0.2	3–50 × 10^3^	1	0.002 to 0.03	Estimated at 1–4% of mRNA by weight	Mortazavi et al. (2008); Ramsköld et al. (2009), Menet et al. (2012)

*The size for the average unspliced pre-mRNA is 17 kb; however, most pre-mRNAs are partially spliced at any given time, and the average size of hnRNA is estimated at 10 kb (Salditt-Georgieff et al., 1976).

**Based on the finding that 1–2% of all mRNA species generate circular RNA, which is present at 10% of the level of the parental mRNA.

By general convention, most other ncRNAs longer than 200 nucleotides, regardless of whether or not they have a known function, have been lumped together into a category called “long non-coding RNAs” (lncRNAs). As a whole, these are present at levels that are two orders of magnitude less than total mRNA (**Table 1**). Although the estimated number of different types of human lncRNAs has ranged from 5,400 to 53,000 (**Table 2**), only a small fraction have been found to be present at levels high enough to suggest that they have a function. According to ENCODE’s own estimates, fewer than 1,000 lncRNAs are present at greater than one copy per cell in the typical human tissue culture cell line (Djebali et al., 2012; Palazzo and Gregory, 2014), although some other estimates have determined that the levels may be substantially higher (Hangauer et al., 2013). One caveat with the data collected thus far is that some of these lncRNAs may have a very restricted expression pattern; therefore until the relevant cell type is tested, we may not be in a position to judge whether it is expressed at a sufficient level to provide evidence of functionality. It is also worthwhile noting that certain annotated lncRNAs may actually encode short functional peptides (Ingolia et al., 2011, 2014; Magny et al., 2013; Bazzini et al., 2014), although in general lncRNAs are poorly translated (Bánfai et al., 2012; Guttman et al., 2013; Hangauer et al., 2013). Finally, it is also worth pointing out that a significant fraction of these lncRNAs may actually be misannotated untranslated regions of known mRNAs (Miura et al., 2013).

**Table 2:** Estimate number of human ncRNAs from various sources.

Source	Number of types	Percent of human genome	Estimated average size (kb)	Reference
Mammalian lincRNome	53647	2.3	1.4	Managadze et al. (2013)
LNCipedia (as of March 2014)*	21487	0.67	1	Volders et al. (2013)
FPKM > 1 lincRNAs	35585	1.1**	NA	Hangauer et al. (2013)
Gencode v7 catalog of human ncRNAs	9277	0.29**	Median of 0.6***	Derrien et al. (2012)
LncRNAs – Gencode v21 (as of 2014 November)	15877	0.50**	NA	http://www.gencodegenes.org/stats.html
Jia et al. (2010)	5446	0.17**	NA	Jia et al. (2010)
Cabili – low confidence	8195	0.26	1	Cabili et al. (2011)
Cabili – high confidence	4273	0.26	1	Cabili et al. (2011)
Small ncRNAs – Gencode v21 (as of 2014 November)****	9534	0.045	0.15	http://www.gencodegenes.org/stats.html
eRNAs	43011	0.34	0.25	Andersson et al. (2014)

*Splice variants were excluded.

**Assumes an average length of 1 kb.

***Median size of 592 nucleotides but with a significant fraction at higher sizes.

****Contains snRNAs, snoRNAs, rRNAs, mitochondrial tRNAs and rRNAs, miRNAs, and “miscellaneous RNAs.”

Other short ncRNAs have been lumped into several groups, depending on their attributes. For example, several regions of the human genome that are believed to be enhancers, are transcribed into short enhancer RNAs (eRNAs). These are thought to act as scaffolds that regulate the 3D architecture of chromosomes in the vicinity of their transcription site (Lai et al., 2013). eRNAs are typically present at even lower levels than lncRNAs (Djebali et al., 2012; Andersson et al., 2014); however, if these play a localized structural role, then they would be expected to be present at only a few copies per cell.

There are still other more exotic species of RNAs (Cech and Steitz, 2014), including circular RNAs (Wilusz and Sharp, 2013). Due to their lack of free 5′ or 3′ ends, circular RNAs are quite stable and some can accumulate to levels that are comparable to mRNAs (Salzman et al., 2012; Jeck et al., 2013; Memczak et al., 2013). However, it is likely that besides a few examples, circular RNAs represent a minute fraction of the total pool of cellular RNAs (see **Table 1**).

In addition to all of the mentioned species, ENCODE and other groups have found transcripts that map to the rest of the genome termed “intergenic RNA” (Djebali et al., 2012). Most of these transcripts are present at levels that are significantly below one copy per cell (Djebali et al., 2012; Palazzo and Gregory, 2014). Again this arbitrary division of ncRNAs has led to much confusion. It is unclear why these transcripts are considered to be intergenic if they are also functional (as in 80% of the genome is functional); after all, if a region of DNA that is transcribed into a functional product is called a gene, then the term intergenic would automatically imply that these regions have no function.

Regardless of these concerns, it is clear that most of the ncRNAs in question (lncRNAs, eRNAs, circular RNAs, intergenic RNAs, etc.) are typically present at very low levels when compared to known functional RNAs. These observations are consistent with the idea that the eukaryotic genome produces a vast amount of spurious transcripts.

## WHERE DO ALL THESE ncRNAs COME FROM?

As of spring 2014, the LNCipedia website^1^ (Volders et al., 2013) has compiled a list of ∼21,000 human lncRNAs, with an average length of about 1 kb (**Table 2**). These would originate from <1% of the human genome. Needless to say, this is a very small fraction of the total. Even if we compiled all of the putative lncRNAs using the most optimistic analysis (Managadze et al., 2013), all the putative lncRNAs would still be transcribed from at most 2% of the genome (**Table 2**). Thus far, only a small minority of lncRNAs have been shown to be important for organismal development, cell physiology, and/or homeostasis. As of December 2014, the LncRNA Database^2^, a repository of lncRNAs “curated from evidence supported by the literature,” lists only 166 biologically validated lncRNAs in humans (Quek et al., 2014). Additionally there are so called eRNAs, which according to FANTOM5 come from an additional 43,000 loci. However, at an average length of ∼250 nucleotides they would be made from ∼0.34% of the human genome (Andersson et al., 2014). Again, these are very small numbers.

In summary, our best candidates for novel functional ncRNAs (lncRNAs, eRNAs) arise from only a minute fraction of the genome. Again it appears that the vast majority of the genome that falls outside of these loci is transcribed into junk RNA that is present at very low levels at steady state.

## BIOCHEMICAL SUPPORT FOR JUNK RNA

It is important to recognize that the pervasive transcription associated with the human genome is entirely consistent with our understanding of biochemistry. Although RNA polymerases prefer to start transcription at promoter regions, they do have a low probability of initiating transcription on any accessible DNA (Struhl, 2007; Tisseur et al., 2011). Indeed it has been observed that most nucleosome-free DNA is transcribed *in vivo* (Cheung et al., 2008) and that many random pieces of DNA can promote transcription by recruiting transcription factors [TFs; see figure S4 in White et al. (2013)].

Of course eukaryotic cells limit the amount of inappropriate transcription by packaging intergenic regions into heterochromatin. This shields the DNA from both RNA polymerases and TFs which can bind to DNA and activate adjacent cryptic transcriptional start sites. The formation of these heterochromatic regions is largely dictated by a complicated array of DNA elements that initiate and restrict chromatin packing. However, there is quite a bit of data that supports the notion that heterochromatin formation is not always strictly regulated or enforced. For example, it has been shown that many heterochromatic regions are transcriptionally active, albeit at a low level (Moazed, 2009), suggesting that either heterochromatin is periodically loosened, or that under certain circumstances RNA polymerases can transcribe these tightly packed regions. Another line of evidence that suggests that heterochromatin formation is not strictly regulated comes from the investigation of TF binding sites. In particular, it has been observed that most TF binding sites which are occupied by TF proteins are not conserved between highly related species (Paris et al., 2013) and that many TF binding events have little to no impact on the expression of nearby genes (Li et al., 2008; Biggin, 2011; Lickwar et al., 2012; Paris et al., 2013). In other words, many putative TF binding sites are created and destroyed by neutral evolution and do not appear to contribute to the expression of functional parts of the genome. These TF binding sites are nonetheless accessible to TF proteins, and thus are not found in heterochromatin.

From the above discussion it is clear that there are many sources for cryptic transcription in eukaryotic genomes. Consistent with this idea, it was found that nascent RNA polymerase II transcripts from mouse liver cells generate a fair amount of transcripts that map to unannotated genomic regions (Menet et al., 2012). When these nascent transcripts were analyzed by next generation sequencing, the number of reads that mapped to intergenic regions (i.e., unannotated parts of the genome) was equal to those mapping to known exonic regions (Menet et al., 2012). Thus it appears that transcription in mammalian cells is quite non-specific.

Although many newly synthesized transcripts likely originate from non-functional parts of the genome, these RNAs are present at very low levels at steady state (Ramsköld et al., 2009; van Bakel et al., 2010; Menet et al., 2012), suggesting that they are rapidly degraded. It is likely that various quality control mechanisms degrade any RNA that lacks features that are overrepresented in either protein-coding mRNAs or other functional ncRNAs (Palazzo and Akef, 2012; Palazzo et al., 2013). This idea is supported by numerous studies that have documented that the level of these spurious transcripts increase when RNA degradation machinery is either depleted or inhibited (Wyers et al., 2005; Davis and Ares, 2006; Thiebaut et al., 2006; Chekanova et al., 2007; Milligan et al., 2008; Preker et al., 2008; Vasiljeva et al., 2008; Neil et al., 2009; Xu et al., 2009; Tisseur et al., 2011; Tan-Wong et al., 2012). It is also supported by the fact that features found in mRNAs (e.g., strong splice sites, polyadenylation sites, etc.) act to stabilize the RNA (Lu and Cullen, 2003; Palazzo et al., 2007; Akef et al., 2013).

Thus it appears that transcription in eukaryotes is very messy, but that much of the junk RNA is removed by quality control mechanisms. This view is completely in line with what is known about the biochemistry underlying eukaryotic gene expression.

## EVOLUTIONARY SUPPORT FOR JUNK RNA

Ultimately to understand how TF binding sites, heterochromatin domains, and transcriptional start sites are created and destroyed within the genome, one needs to take into consideration certain concepts that have been derived from the field of population genetics.

One of the most fundamental discoveries in population genetics came from the work of Kimura, Ohta, King and Jukes. They showed that the ability of natural selection to weed out slightly deleterious mutations depends on the size of the breeding population in a given species (Kimura, 1968, 1984; King and Jukes, 1969; Ohta, 1973). The higher the number of individuals, the more powerful natural selection is at identifying slightly deleterious mutations and eliminating them. Due to certain aspects of population dynamics, the effective population size is far smaller than the number of individuals [for a more detailed discussion see (Lynch, 2007)]. For modern humans, the effective population size has been calculated to be 10,000 throughout most of its history, which is typical for mammals (Charlesworth, 2009). Indeed there exists an inverse linear correlation between the effective population size and how deleterious a mutation has to be before it can be effectively eliminated from a population by natural selection. In the absence of selection pressure, some neutral and slightly deleterious mutations will reach fixation due solely to genetic drift [for an extensive examination of this process see (Lynch, 2007)]. It is also important to realize that this relationship also applies to slightly beneficial mutations – there is an inverse correlation between the effective population size and how beneficial a mutation has to be before it can be effectively selected for by natural selection. Thus when one observes some genetic alteration, it is critical that we keep in mind how the alteration affects the fitness of the organism and whether this change can be acted on by selection (either positively or negatively) given the size of the population.

Given that the displacement of a few nucleosomes can promote transcription initiation (Cheung et al., 2008), that TF binding sites and transcriptional start sites are made up of small degenerate sequences (Stewart et al., 2012), and that many random pieces of DNA can activate transcription (White et al., 2013), we would expect that a large number of random mutations would create fortuitous transcriptional start sites. Importantly, natural selection will be powerless to prevent the appearance of these sites, as long as the resulting RNA is not too deleterious to the organism. Conversely, a transcriptional event needs to provide a substantial advantage before natural selection can act to preserve this alteration in future generations. Most of the data on eukaryotic genomes support the view that the fixation of most genomic alterations are due to drift, while few can be ascribed to positive selection (Lynch, 2007).

Thus the presence of a certain level of junk RNA is not only compatible with our understanding of evolution, but would be expected. Nevertheless, it still remains unclear how much junk RNA a eukaryote could tolerate before natural selection would begin to eliminate it.

## THE DANGERS OF HYPERADAPTATIONISM

The overreliance on adaptationist “just-so stories” in the field of evolutionary biology has been openly criticized since the 1970s. Famously, Gould and Lewontin (1979) compared such thinking to the ideology espoused by Pangloss, the fictional professor from Voltaire’s novel Candide who used just-so stories to prove that we lived in the best of all possible worlds. Unfortunately hyperadaptionalism, or the belief that the vast majority of traits found in an organism (including its DNA) are present due to some selective force, has plagued much of molecular biology as well (Sarkar, 2014). The proclamation that a biochemical activity is equivalent to function (ENCODE Project Consortium et al., 2012) is just another example of this ideology. Using this logic we would state that any transcribed DNA is functional, but would this mean that the transcript (or transcriptional process) is functional by virtue of its mere existence? To resolve this paradox, we would either have to state that (1) although the DNA is functional, its output, the RNA (or the act of transcription) is not; or (2) that all RNAs are *de facto* functional. Obviously both of these nonsensical conclusions have their roots in hyperadaptionalist thinking and an abuse of the concept of biological function. To resolve this, we need to install a more rigorous definition of function. However, this can only be accomplished if we properly define the null hypothesis.

## THROWING DOWN THE GAUNTLET: THE HYPOTHETICAL EXAMPLE OF A NON-FUNCTIONAL ncRNA

To determine the degree to which a process is adaptive, it is important to establish how the exact same events would evolve by non-adaptive mechanisms. Selection should only be invoked when non-adaptive explanations do not suffice. This viewpoint has been used to determine the contribution of selection to alternative splicing, RNA editing and in determining the lengths of UTRs and introns (Lynch, 2007; Huang and Niu, 2008; Wang et al., 2014; Xu and Zhang, 2014). Here, we would like to introduce the example of a hypothetical non-functional ncRNA as a useful null hypothesis. Again, adaptation (and hence function) should only be invoked if an ncRNA has more attributes than our hypothetical non-functional ncRNA. Using principles of biochemistry and population genetics, we will describe its attributes.

### EXPRESSION LEVELS

This putative non-functional ncRNA would be present at levels that would not be a burden to the cell. There are three considerations to take into account when considering the level at which a ncRNA is present.

First the mere presence of the ncRNA may act as a burden. The typical mammalian tissue culture cell has on the order of 500,000 mRNA molecules. Other RNAs with unknown function (i.e., “intergenic” RNA and lncRNA) are at levels between 1 and 4% those of mRNA (Mortazavi et al., 2008; Ramsköld et al., 2009; Menet et al., 2012) and thus present on the order of about 10,000 total copies per cell (**Table 1**). Therefore if a hypothetical ncRNA were present at 10 copies per cell at steady state, they would increase the pool of intergenic/lncRNAs by 0.1%, and would increase the total pool of RNA by a negligible amount (**Figure 1**).

Second, there is a cost to synthesizing the RNA. One study that investigated the energetics of synthesizing long introns has estimated that for an mRNA that is expressed at a level of 30 copies per cell and a half-life of 1 h (resulting in the generation of 360 new RNA molecules/cell per day), an intron would have to be roughly 83,000 nucleotides long for it to be a significant burden, given the effective population size of humans (Huang and Niu, 2008). Using these figures, we can estimate that in humans a non-functional ncRNA that is 1 kb in length and is ubiquitously expressed throughout the body would have to be synthesized at a rate of almost 30,000 copies per cell per day before it would be eliminated by natural selection. Of course if the ncRNA was spliced from a longer transcript, this number would be less.

Third, the ncRNA may have some associated activity that may be deleterious. Most often the major concern is whether it will be translated into short random peptides (see point 3, below). Although ncRNAs are poorly translated, most studies have found that they can be engaged by the ribosome at low levels (Guttman et al., 2013). This can be further mitigated by subcellular localization (see below). Thus as long as the putative ncRNA does not have some activity that negatively impacts some cellular function or the organism in general, our guess would be that if a ncRNA was present even at a level of 10 copies per cell, this small increase in the ncRNA burden would be tolerable (i.e., not deleterious enough to be subjected to negative selection).

### EXPRESSION PROFILES

We might imagine that our putative non-functional RNA was transcribed due to the fortuitous action of one or more TF binding events. As described above, it is likely that many such sites exist in the mammalian genome as the number of active transcriptional start sites exceed the number of protein-coding genes by an order of magnitude (Carninci et al., 2006). Since the majority of TFs are expressed in a developmentally or spatially regulated manner, it follows that our hypothetical ncRNA will also be expressed in a manner that appears to be under some sort of precise regulatory control. Some researchers have tried to claim tissue-specific expression patterns provide some proof of functionality (Ponting et al., 2009; Hangauer et al., 2013; Mattick and Dinger, 2013); however, such a restricted expression of the ncRNA is entirely consistent with a lack of function.

### DISTRIBUTION OF THE ncRNA IN THE CELL

In determining how our putative non-functional RNA would be distributed intracellularly, there are several facts to take into account. First, if this RNA were to be exported to the cytoplasm, it is reasonable to believe that it would be a substrate for the translational machinery, as long as the RNA is free of extensive secondary structures. Second, this RNA would be translated into a random polypeptide. Unlike nucleic acids, unstructured polypeptides have a high tendency to aggregate and activate cellular stress (West et al., 1999; Chi et al., 2003). Lastly, a single RNA molecule can be used to generate many polypeptides, thus amplifying any potential deleterious effects. For these reasons, we believe that non-functional RNAs are much more likely to promote cellular stress if they are present in the cytoplasm where they can be translated by ribosomes. Indeed, it is likely that the nucleo-cytoplasmic division evolved in part to prevent ribosomes from translating misprocessed mRNAs and aberrant RNA transcripts (Martin and Koonin, 2006; Akef et al., 2013; Palazzo and Gregory, 2014). This may be the reason that features associated with mRNAs tend to promote their nuclear export (Palazzo and Akef, 2012), while problems during translation will promote the degradation of the RNA by processes such as non-sense mediated decay (Baker and Parker, 2004). These reasons may explain why most lncRNAs are nuclear (Derrien et al., 2012; Djebali et al., 2012) and not significantly translated (Guttman et al., 2013). By this same logic we would expect that our putative ncRNA would not likely be present in the cytoplasm, although we do not yet have any hard data about what level of cytoplasmic ncRNA would be tolerable.

From this discussion it makes sense that our non-functional ncRNA would be nuclear, but what about its localization to a specific sub-nuclear compartment? Again, some have used localization to sub-nuclear loci as proof of functionality (Mattick et al., 2010; Kapusta and Feschotte, 2014). In experiments performed in our lab we have documented how reporter RNAs with an essentially random sequence are indeed localized to discrete nuclear foci. In some cases these colocalize with known nuclear structures, such as nuclear speckles (Akef et al., 2013); in other instances these RNAs form discrete nuclear puncta that are of unknown nature (Lee and Palazzo, unpublished observations). These observations suggest that even sub-nuclear compartmentalization cannot be used as evidence to support functionality for any ncRNA.

### PROCESSING

We would expect that the non-functional ncRNA would lack strong processing signals, as these regions would be expected to be under strong purifying selection only in functional spliced transcripts. For example in most mRNAs, introns are not only flanked by splicing donor and acceptor sites but are also defined by location of intronic and exonic splicing elements (Blencowe, 2000; Wang et al., 2012). However, to our knowledge, no one has systematically studied the splicing of randomly generated RNAs. Despite this we can still estimate the prevalence of splicing signals computationally. For example, the occurrence of consensus donor and acceptor splice sites in essentially random human DNA sequences are one every 3 and 10 kb, respectively (Shepard et al., 2009). Because the spliceosome requires suboptimal sequences to initiate splicing, it is likely that the actual number of potential donor and acceptor sites is much higher. Thus if the primary transcript of our non-functional RNA is long enough, it will probably be spliced to a certain extent. As for smaller transcripts, a small but significant number are also likely to be spliced. However, since splicing helps to stabilize the RNA (Palazzo and Akef, 2012), it is likely that a non-functional ncRNA would only be present at detectable levels by virtue of the fact that it is spliced. In other words, although a lack of processing would lead to the instability of many functionless RNAs, we would expect that a small minority of junk RNAs would be spliced and hence stabilized, and it is precisely these ncRNAs that would be under investigation.

Polyadenylation signals are also likely to be present in our putative junk RNA. These sites are quite abundant – to the extent that many of these sites are present in introns but are normally suppressed by the action of the spliceosome. These cryptic 3′cleavage sites become quite heavily used in cells with reduced U1 snRNA levels (Kaida et al., 2010). As with splicing, polyadenylation promotes mRNA stability (Akef et al., 2013); thus many junk RNAs that would be present at detectable levels are likely present by the very fact that they are polyadenylated. In summary, the fact that a given ncRNA is spliced and polyadenylated is entirely consistent with it not having any function.

Certain groups, such as the HUGO Gene Nomenclature Committee (Wright and Bruford, 2011), have defined lncRNAs as being “spliced, capped and polyadenylated,” with the clear implication that these processes are more likely to be found in functional RNAs than stable junk RNA. We disagree with this view on three counts. First, some non-functional RNAs may be processed [as described above, and by others (Ulitsky and Bartel, 2013)]. Second, many known functional ncRNAs lack all of these processing steps, one example being 7SL (Walter and Blobel, 1982; Ullu and Weiner, 1984). Third, although the goal of this nomenclature is presumably to identify functional non-coding RNAs, as is implied by the term “lncRNA,” these groups never come out and categorically state whether they consider these RNAs functional (although we assume that they do). If the term lncRNA does not imply function, then what exactly does it mean? Is it a meaningless term?

The important distinction between functional and non-functional RNAs is that processing signals are under a high level of selection pressure in the former but not in the latter. Thus, although non-functional ncRNAs may be processed, they will likely have weak signals. Interestingly, introns in lncRNAs tend to be spliced post-transcriptionally, while those in mRNAs tend to be removed co-transcriptionally (Derrien et al., 2012; Tilgner et al., 2012). This may suggest that many lncRNA introns have weak signals due to a lack of selection pressure. Of course a paucity of hard data about the processing of random RNA polymers prevents us from making firmer conclusions. Perhaps studies such as the random genome project (Eddy, 2013) would help us identify how often spliced non-functional ncRNAs would occur purely by chance from a given stretch of DNA.

### CONSERVATION

It has been demonstrated for the last 50 years that sequence conservation is a reliable indicator of function. In line with this thinking, many commentators have declared that conservation should be the only criterion for identifying functional genomic loci (Doolittle et al., 2014). In agreement with this, we would expect that our non-functional ncRNA would accumulate mutations at a rate consistent with genetic drift. Indeed some groups have tried to restrict their definition of lncRNAs by using conservation (Guttman et al., 2009).

There are, however, various circumstances that may give the appearance of conservation. For example, the transcribed loci may also contain some conserved functional element, such as a critical TF binding site. If the region of conservation is confined to a pseudogene or TE sequences, one may simply be detecting these entities, which are typically non-functional.

The other problem with relying exclusively on sequence conservation to define functionality is that we know of many genomic loci which have sequence-independent roles. In many cases these regions serve as spacers. Thus natural selection may conserve the presence of any sequence, but not a precise sequence. For example, 5′UTRs and introns need to have a minimal length in order to promote robust translation initiation (Kozak, 1991) and splicing (Wieringa et al., 1984), respectively. Other examples include centromeric-associated repeats, which serve as sequence-independent scaffolds for kinetochore assembly (Torras-Llort et al., 2009). It is also possible that certain ncRNAs may act as a sequence-independent scaffold for protein-binding, as likely is the case of the regulation of HP1 by transcripts produced from heterochromatic regions of the *Saccharomyces cerevisiae* genome (Keller et al., 2012). Some evidence exists supporting the idea that certain eRNAs may recruit the Mediator complex to form DNA-loops, and this may require very little sequence specificity in the RNA itself (Lai et al., 2013; Andersson et al., 2014; Shibayama et al., 2014). Other times, the act of transcription, and not the resulting ncRNA, may play a role in regulating the expression of nearby genes. Presumably, the initiation of these putative regulatory transcription events are due to the activity of transcriptional start sites and/or other critical *cis*-acting elements that do display some degree of conservation. However, in practice, these promoters may be hard to identify solely by sequence analysis.

There has also been much talk about human-specific functional ncRNAs, which have generated considerable interest since they could potentially help explain differences between us and related species (Wu et al., 2013). Although these ncRNAs would not be conserved between species, they could in principle be distinguished from non-functional ncRNA by the analysis of numerous human genomes. We would predict that non-functional ncRNA would diverge between individuals within the species at a rate comparable with genetic drift. In contrast, loci producing functional ncRNAs would be conserved. This calculation would depend on when the region in question became fixed and how fast it spread in the population. Unfortunately determining these parameters is not straight forward as it requires a large number of human genomes to be sequenced. Further, complicating the issue is the possibility that the ncRNA locus in question might be located near a genomic region that was under positive selection. The spread of neutral loci by riding on the coattails of nearby positive mutations is known as hitchhiking or draft and may be quite common (Gillespie, 2000). For these reasons, sorting lineage-specific functional ncRNA genes from non-functional ncRNAs is not trivial. Even when one turns to protein-coding genes, many of those that were once thought to be human-specific may not code for proteins after all and may indeed be non-functional (Ezkurdia et al., 2014). It is useful to keep in mind that if our ability to spot lineage-specific coding genes is problematic and fraught with error, the identification of functional human-specific ncRNAs would be even more difficult.

### CAUSAL ROLES

As stated above, certain commentators have championed selection as the primary arbiter of whether a genomic locus is functional. These same individuals have dismissed any evidence that is based on causal roles, which is defined as “the way(s) in which a component contributes to a stated capacity of some predefined system of which it is a part: what it in fact does” (Doolittle et al., 2014). The problem with defining functionality with causal roles, according to these commentators, is that this concept can be easily misappropriated. For example, a given genetic locus may be transcribed (i.e., caused the production of an RNA), but this event may not necessarily contribute to the fitness of the organism. Only if this activity was important, then natural selection would act to conserve it. Thus in the absence of any evidence of selection, regions of the genome that display some sort of causal role are *likely* not functional. This is not an absolute statement. As we point out in the previous section, certain functional RNAs may have a critical role that is sequence-independent. In other circumstances, the act of transcription, and not the ncRNA (or presumably its sequence), plays some critical role.

In light of these problems, the question clearly becomes, can a non-functional ncRNA be distinguished from one that is functional, simply on the basis of an experiment that demonstrates a “causal role”? In our opinion the answer is yes, as long as the appropriate causal role is chosen. By definition, elimination of functional ncRNAs should affect homeostasis, development or other important biological processes that would impact the fitness of the organism. In contrast, other causal role events that could potentially be associated with non-functional ncRNAs would be insufficient to qualify as evidence of functionality.

There are some problems with relying on causal roles to determine function, in that it is not always clear whether an activity could occur by chance with an RNA with a random sequence. For example, if the overexpression of an ncRNA promotes oncogenesis, would this provide evidence of functionality? This hypothetical ncRNA could simply be sequestering an RNA binding protein that has a pro-apoptotic function, and in this instance this type of evidence would be weak. If, on the other hand, the ncRNA in question acted as a ribozyme that generated free radicals which caused DNA damage, this would then be much stronger evidence, as this activity would not be expected from a random RNA. Other evidence, such as the association of lncRNAs with certain protein complexes [e.g., the polycomb repressive complex (Khalil et al., 2009)], seems unclear. How often would such an association occur with a random non-functional nuclear RNA?

Ultimately, the ideal experiment is to determine whether the elimination of an ncRNA affects a biological process that is required for the proper development or homeostasis of the organism. This has become more feasible with the advent of CRISPR/Cas9 technology (Doudna and Charpentier, 2014). One serious problem with this approach is that the elimination of a given ncRNA may only have a small impact on the biological process being assessed and thus results in a small reduction in fitness, for example, reducing the number of offspring by 0.1%. Such small effects would be hard to detect in a laboratory setting but would be strongly selected against in the wild, and would indicate that the RNA has a function. In this case it might be beyond our current experimental abilities to obtain causal evidence for certain functional ncRNAs.

## BUILDING A CASE FOR FUNCTION

To date, projects such as ENCODE, LNCipedia and the HUGO Gene Nomenclature Committee have distinguished lncRNAs from junk RNA primarily based on expression levels and RNA-processing. In contrast, we believe that researchers need to evaluate whether any putative functional ncRNAs have properties that are beyond what one would expect from a non-functional ncRNA, given our knowledge of biochemistry, genomic evolution and current empirical data. Evidence for function can consist of expression levels that are very high (i.e., imposing a significant cost on the organism), a high degree of conservation, and/or experimental evidence that the ncRNA is required for some important biological process. Importantly, ncRNAs should be evaluated on a case-by-case basis. In the absence of sufficient evidence, a given ncRNA should be *provisionally labeled as non-functional*. Subsequently, if the ncRNA displays features/activities beyond what one would expect for the null hypothesis, then we can reclassify the ncRNA in question as being functional.

## CONCLUSION

It is clear that the human genome contains a large number of functional ncRNAs. Indeed it is likely that the list of biologically validated ncRNAs, as listed in the LncRNA Database (Quek et al., 2014), will continue to grow. As others have pointed out, even if 10% of current lncRNAs prove to be functional, this would represent a wealth of new biology. However, given our current understanding of biochemistry and evolution, it is likely that most of the RNAs generated from the low levels of pervasive transcription, and likely a substantial number of currently annotated “lncRNAs,” are non-functional.

## Conflict of Interest Statement

The authors declare that the research was conducted in the absence of any commercial or financial relationships that could be construed as a potential conflict of interest.

## Footnotes

1 http://www.lncipedia.org/

2 http://www.lncrnadb.org/
